# Skin Lesion Classification Using Collective Intelligence of Multiple Neural Networks

**DOI:** 10.3390/s22124399

**Published:** 2022-06-10

**Authors:** Dan Popescu, Mohamed El-khatib, Loretta Ichim

**Affiliations:** Faculty of Automatic Control and Computers, University POLITEHNICA of Bucharest, 060042 Bucharest, Romania; mohamed.el@stud.acs.upb.ro (M.E.-k.); loretta.ichim@upb.ro (L.I.)

**Keywords:** skin lesions classification, convolutional neural networks, data augmentation, residual blocks, dense blocks, inception module, multi-networks system, data fusion, decision weight, collective intelligence

## Abstract

Skin lesion detection and analysis are very important because skin cancer must be found in its early stages and treated immediately. Once installed in the body, skin cancer can easily spread to other body parts. Early detection would represent a very important aspect since, by ensuring correct treatment, it could be curable. Thus, by taking all these issues into consideration, there is a need for highly accurate computer-aided systems to assist medical staff in the early detection of malignant skin lesions. In this paper, we propose a skin lesion classification system based on deep learning techniques and collective intelligence, which involves multiple convolutional neural networks, trained on the HAM10000 dataset, which is able to predict seven skin lesions including melanoma. The convolutional neural networks experimentally chosen, considering their performances, to implement the collective intelligence-based system for this purpose are: AlexNet, GoogLeNet, GoogLeNet-Places365, MobileNet-V2, Xception, ResNet-50, ResNet-101, InceptionResNet-V2 and DenseNet201. We then analyzed the performances of each of the above-mentioned convolutional neural networks to obtain a weight matrix whose elements are weights associated with neural networks and classes of lesions. Based on this matrix, a new decision matrix was used to build the multi-network ensemble system (Collective Intelligence-based System), combining each of individual neural network decision into a decision fusion module (Collective Decision Block). This module would then have the responsibility to take a final and more accurate decision related to the prediction based on the associated weights of each network output. The validation accuracy of the proposed system is about 3 percent better than that of the best performing individual network.

## 1. Introduction

Segmentation and classification of skin lesions in the early stages is very important for the detection of possible malignancies. In this case, the malignant lesions can be treated with a high chance of cure; otherwise, they can lead to metastases and death. It is very important to obtain highly accurate systems when it comes to skin lesion diagnosis since some could be found to be a melanoma, which is one of the deadliest types of cutaneous cancer. Currently, melanoma prevention and detection in early stages represent an important aspect and concern to specialists. The number of cancer cases is estimated to increase by 24.1% for men and 20.6% for women in the following years [1]. A very high incidence of annual cancer cases can be seen on the skin. There were about 100,000 cases in 2020 in the United States out of which about 10,000 were fatal [2]. Indeed, compared with other types of cancer, the mortality rate is not high (10%). However, patient treatment is often painful and if the disease is not detected in time, it can end in the amputation of the affected area. Skin cancers are divided into basal cell carcinoma, squamous cell carcinoma, Merkel cell carcinoma, and melanoma. Out of these four types, melanoma represents the most severe form, with the highest degree of mortality. A systematic review concerning melanoma detection by neural network-based systems was done in [3].

Image processing for the detection, segmentation, and classification of skin lesions encounters difficulties such as [4]: (a) location in different areas of the body, size, and shape; (b) the existence of noise and artifacts (hair, bubbles, and blood vessels); (c) irregular, random and sometimes diffuse edges (low contrast between the lesion and healthy skin); (d) faulty lighting; (e) images are taken by types of equipment with different characteristics. Therefore, image preprocessing is always necessary to improve them before a proper analysis.

Comparing the average performances of predictions of skin lesions in photographic or dermoscopic images by dermatologists with those by deep learning convolutional neural networks found that the latter outperforms the former [5]. Because of this, automated diagnosis systems based on neural networks can be used as decision support systems in the detection and diagnosis of skin lesions, especially in malignant cases (and most importantly, in the case of melanomas). Of course, each dermatologist has his own opinion and therefore, it can be correct or wrong when it comes to providing a diagnosis. Therefore, as in the case of specialists, from our point of view, it is very important to obtain more opinions from more neural networks and to conclude a final output/decision/prediction.

This paper aims to present a novel system, able to classify different types of skin lesions, including melanoma, based on the output of multiple individual convolutional neural networks, trained on the HAM10000 dataset [6], containing seven different classes of interest (seven possible diagnoses). A decision fusion module based on collective intelligence on multiple neural networks (NNs) is considered a global classifier. It takes into consideration the weighted output of each of the selected neural networks and will act as a maximum decision-based system. The weight, associated with each NN output and individual predicted class, is calculated based on individual performances. The proposed system is more accurate than each of the individual NNs since it is meant to simulate a common behavior in real life, where a specific problem (disease) is solved by multiple specialists, each of them providing an estimation/prediction/solution based on their accumulated experience over the years and all outputs are then collected globally, gaining advantages from each to provide a final output.

This paper involves multiple CNNs pretrained either with an ImageNet dataset or Places-365 dataset, trained on HAM10000, and configured in such a manner to obtain better performances (in terms of accuracy), even if we are talking this time about seven classes of interest, instead of only two classes (melanoma vs. common nevi), which are commonly investigated already in the literature. Moreover, another difference is related to the number of data samples used in training (10,015 images), instead of a smaller number.

Next, the related works in the same field are investigated. The paper continues with the materials and methods used for designing the proposed skin lesion classification system. Thus, we present the necessary pre-requisites for data acquisition, preparation, augmentation, an overview of the used dataset, and all CNNs used in our experiments: AlexNet (NN_1_), GoogLeNet (NN_2_), GoogLeNet-Places365 (NN_3_), MobileNet-V2 (NN_4_), Xception (NN_5_), ResNet-50 (NN_6_), ResNet-101 (NN_7_), InceptionResNet-V2 (NN_8_), DenseNet201 (NN_9_), and our proposed solution based on collective intelligence: Collective Intelligence System (CIS). We then continue by presenting the experimental results for each of the involved networks and those for the CIS. The CIS performances are better than each NN_i_ performance. Finally, the discussion section and conclusion are presented.

## 2. Related Works

According to the latest research papers in this field [3], the tendency is to design skin lesion systems using different techniques:

- Standard techniques/other classifiers such as combining ABCDE with SVM.
- One modified network and trained via a transfer learning technique.
- Multiple networks which are, in general, either combined in one global classifier or combined in a series ensemble (e.g., one network realizes segmentation and one uses the output for classification).
- Other classifiers combined with one or multiple networks.

Today, deep learning is widely used when it comes to skin lesion detection and diagnosis. It was demonstrated over time that good results were obtained using the CNNs that we chose to experiment with within our collective intelligent system. Even if AlexNet [7] is an old state-of-the-art CNN, it was already used and continues to be used in different experiments, when it comes to the skin lesions field. In [8] was obtained an accuracy of 93.64% for a skin mole detection system using AlexNet to extract features together with KNN for classification. It can be seen that combining multiple methods was already a trend.

Esteva et al. [5] proposed a system that performs as well as dermatologists when it comes to identifying malignant lesions. The authors used a GoogLeNet Inception-V3 NN and trained it using 129,450 skin lesion images to be able to classify skin lesions as benign/malignant. The output of the system was compared with the performance of 21 different dermatologists and the result was a positive one.

In time, CNNs were getting deeper and thus the networks were vanishing gradient issues. Therefore, for deeper and more accurate models, researchers within the skin lesions field started using residual networks to overcome the vanishing gradient problem. ResNet-50 and ResNet-101 are also convolutional neural networks that are widely used for skin lesion diagnosis. ResNet-50 was used in [9] to classify melanoma and nevus. The authors combine handcrafted features such as color, shape, and texture and deep learning features extracted by multiple networks. Mutual information was then used as a fusion rule to obtain the most important aspects from both types of features and multiple classification methods such as Linear Regression, Support Vector Machines, and Relevant Vector Machines. The overall system accuracy in the case of ResNet-50, being used as a deep learning feature extractor, was 90.67%. In the same paper [9], experiments were also done with Xception and MobileNet-V2, two other networks which we took into consideration. In the case of Xception being used as a feature extractor, the authors obtained an overall accuracy for melanoma and nevus classification of 90.47%, while, in the case of MobileNet-V2, they obtained 92.40, being the best performance obtained as compared with other experiments. ResNet-101 is another similar network with good results in skin lesion detection and diagnosis [9,10,11,12]. In the case of [10], it was used as part of an ensemble composed of multiple individual networks combined for obtaining better performances.

DenseNet-201 is also a commonly used convolutional neural network in skin lesion diagnosis research papers. Al-Masni et al. [13] proposed an accurate classification system based on two phases. The first phase is based on skin lesion segmentation using a fully convolutional network and the second phase is based on feature extraction using multiple convolutional networks, including DenseNet-201. The overall system achieved an accuracy of 77.04% accuracy on the ISIC 2016 dataset and 81.29% on the ISIC 2017 dataset.

InceptionResNet-V2 is also widely used in skin lesion diagnosis. For example, authors in [14] propose a solution for learning discriminative features from skin images by “fine-tuning” ResNet-152 and InceptionResNet-V2 layers with a triplet loss function. The overall system accuracy was 87.42% and was represented by the model using InceptionResNet-V2. In this paper, the mentioned NNs were used separately to identify skin diseases such as acne, dark circles, and spots.

In general, according to the latest reviews related to the skin lesions field, researchers tend to use ensemble models for obtaining better results by combining multiple deep learning/machine learning techniques using their own algorithms. One example of such a system is [15], where the authors proposed a framework for an accurate skin lesion classification system with two classes (benign/malignant). The initial step is lesion segmentation considering several methods: contrast stretching, mean segmentation, mean deviation-based segmentation, and image fusion. The segmented images are then passed to multiple pre-trained models such as Inception-V3, InceptionResNet-V2, and DenseNet-201 for feature extraction. Then a features vector is constructed. The next step is represented by a proposed solution for selecting the most significant features and discarding the less significant ones based on entropy-controlled neighborhood component analysis. The output is provided to a KNN classifier to give the final prediction (benign or malignant). Another example of a system using combined decision is [16], where a more complex multi-network voting system was proposed. The system is based on multiple convolutional neural networks, each of them being specialized in performing binary classification of a particular disease and providing a vote/value for a single input image. The ensemble system computes the maximum value from all outputs and compares it with a threshold. In the case of a larger value, the final prediction is the one with the maximum value; otherwise, the responsibility is then passed to a group decision voting module, providing the final decision.

Based on the recent review [3], such ensemble models represent some of the new trends in designing accurate melanoma detection systems. For example, the authors in [10] proposed a melanoma detection system based on a custom NN, GoogLeNet, NasNet-Large, ResNet-101, and a feature-based classifier. The authors also introduced a global classifier where they took a final decision based on the probabilities of individual classifiers, with two classes: melanoma and non-melanoma.

A similar approach, based on a global classifier, could be found in [17], where the authors proposed a system based on six neural networks connected in two operational levels. The first level contains five individual classifiers (LBP + Perceptron, HOG + Perceptron, GAN + ABCD rule, ResNet, and AlexNet) while the second level is being represented by a Perceptron-type classifier with a convolutional layer based on fixed weights, which has the responsibility of taking the final decision (melanoma/non-melanoma).

## 3. Materials and Methods

### 3.1. Dataset Used

There are many datasets with skin lesions: PH2, ISIC 2016, ISIC 2017, ISIC 2018-HAM10000, ISIC 2019, ISIC 2020, DERMQUEST, MED-NODE, DERMNET, DERMIS, DERMOFIT, etc. [3]. HAM10000 is one of the largest skin lesions datasets publicly available for academic research. In this paper, for current experiments, we chose to use the HAM10000 (“Human Against Machine with 10,000 training images”) dataset, introduced in the ISIC 2018 challenge, which contains 10,015 dermatoscopic images which can serve as a training dataset for academic machine learning purposes [6]. The HAM10000 dataset covers image samples for all-important diagnostic categories (classes) in the real pigmented lesions:

- Actinic keratoses and intraepithelial carcinoma/Bowen’s disease (akiec)
- Basal cell carcinoma (bcc)
- Benign keratosis-like lesions (bkl-solar lentigines/seborrheic keratoses and lichen-planus like keratoses)
- Dermatofibroma (df)
- Melanoma (mel)
- Melanocytic nevi (nv)
- Vascular lesions (vasc-angiomas, angiokeratomas, pyogenic granulomas, and hemorrhage).

**Figure 1:**
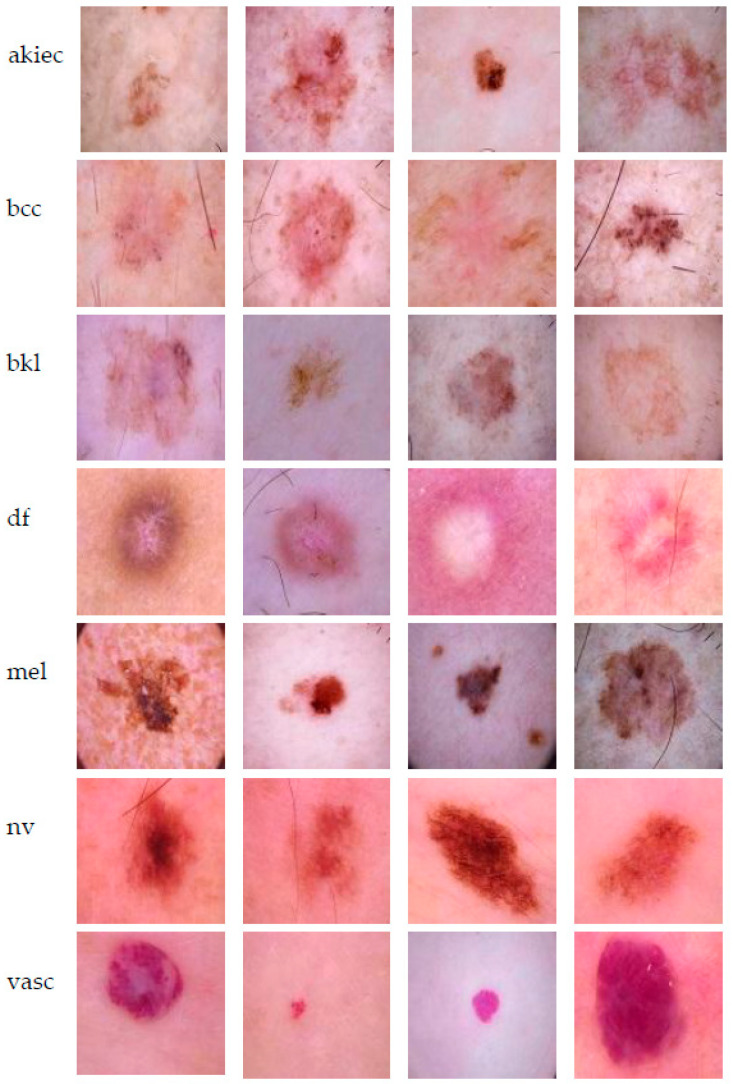
Example of each class from HAM10000 dataset [6].

HAM10000 dataset contains 1015 JPEG images and is split into two packages/folders: HAM10000_images_part1.zip (5000 JPEG images) and HAM10000_images_part2.zip (5015 JPEG images). Some examples are presented below in Figure 1.

Data augmentation can be useful in the training phase if the data in certain classes of the dataset is small. This can reduce the overfitting of the deep neural networks. For example, in [18], the authors propose a two-stage data augmentation framework, one for learning and one for testing in the case of these neural networks.

**Figure 2:**
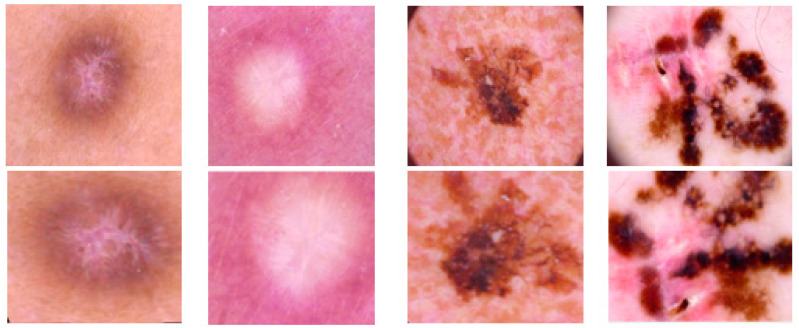
Examples of applied data augmentation.

We used dataset augmentation methods to try to balance the data (classes). As can be seen in Figure 2, the following augmentation methods were applied:

- Shearing, rotations (on both vertical and horizontal axis);
- Mirroring;
- Random image zoom;
- Vertical or horizontal pixel shift with a maximum of 10%.

We observed that the HAM10000 dataset has lots of nevus samples, while other classes are not balanced and have a smaller number of images. Therefore, in the training phase, we chose to augment and obtain more representatives (200 images) for classes with a smaller number of images (melanoma, dermatofibroma, etc.) and remove the same number of images from the nevus class, thus balancing both training and validation datasets.

Obviously, there may be a correspondence between the complexity of the model and the structure of the classes. The closer they look, the more intelligent a system is needed. Moreover, this is the case for a small number of images per class. We associate increased collective intelligence with the number of individual NNs, but as a compromise with time and complexity.

### 3.2. Neural Networks Used

There is a compromise regarding the choice of the number of neural networks. On the one hand, collective intelligence in decision-making seems more credible if there are more subjects. On the other hand, there are difficulties regarding the complexity of the system. The networks were selected based on a literature investigation and on experiments performed by authors on several neural networks. Having gained the results for each, we proposed a method to combine their decisions to globally obtain a better performance as compared with individual ones. The trained networks have a minimum impact when it comes to execution time.

In the proposed system we used 9 NNs considered as individual intelligent classifiers. These networks (AlexNet, GoogLeNet, GoogLeNet-Places365, ResNet-50, ResNet-101, Xception, MobileNet-V2, DenseNet-201, and InceptionResNet-V2) will be the backbone of the collective intelligence system.

AlexNet was firstly introduced in [7] and represents one of the first state-of-the-art CNNs, which, according to [3], was widely used in the classification of skin lesions. The network is composed of 8 layers, out of which 5 are convolutional layers and 3 are fully connected layers. The last fully connected layer has 1000 neurons and since the network was trained using the ImageNet dataset (over one million images), it was able to classify 1000 objects (pencil, keyboard, etc.).

GoogLeNet [19] was implemented by Google researchers in 2014 and was the winner of the ILSVRC 2014 image classification challenge. It is also widely used in skin lesion classification tasks [3]. It represents the first version of the network to introduce the Inception module, which represents the network’s basic block. The 1 × 1, 3 × 3, 5 × 5 convolution blocks, and 3 × 3 max pooling blocks perform in parallel, the output being concatenated and passed to the next layer. The simplified version of GoogLeNet has 22 layers and can classify 1000 classes, being also trained on ImageNet. In the case of our experiments, we chose to use a pre-trained version of GoogLeNet on the Places365 dataset. Based on the obtained results we illustrate the importance of the initial weights of the network and the impact in the final classification after performing the transfer learning technique using the HAM10000 dataset. Therefore, we consider two NNs based on GoogLeNet: one pre-trained on the ImageNet dataset (GoogLeNet) and the other pre-trained on the Places365 dataset (GoogLeNetPlaces365).

**Figure 3:**
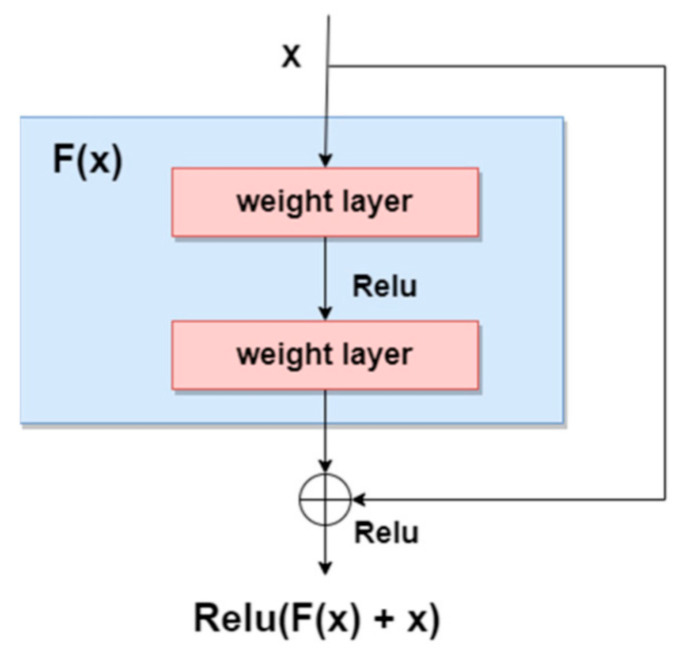
Residual block [3].

As stated in [3], a new trend for designing skin lesions diagnosis systems is to build deeper networks and it was demonstrated that, as the network gets deeper with more layers, the training error would increase over time because of the vanishing gradient problem. Residual networks are meant to solve this issue by proposing the “Residual” block in [20], presented in Figure 3, to provide the ability to be able to design deeper networks and obtain a better accuracy to discover more and more patterns. As we can see, residual blocks sum the result of the previous layer with the result of the applied function (current layer) on the original input, thus also maintaining valuable information.

The residual NN ResNet-50 is one of the most used CNNs in skin lesion diagnosis systems together with other versions: ResNet-101 and ResNet-152. It is a 50-layer deep CNN composed of 48 convolution layers, one max-pooling layer, and one average pooling layer. ResNet-101 is another version of residual networks, but this time, it is a deeper one, being composed of 99 convolution layers, one max-pooling layer, and one average pooling layer. It is a deeper network that is able to learn more patterns and at the same time can also solve the vanishing gradient problem.

Xception is another CNN, often used in skin lesion diagnosis systems [3]. It was initially proposed in [21] and it was meant to over-perform GoogLeNet, by replacing Inception modules with depth-wise separable convolutions. Xception is a deep CNN with 71 layers and it is composed of three main blocks: entry flow, middle flow, and exit flow. Residual connections are also used to solve the vanishing gradient problem.

MobileNets are CNNs usually designed for mobile and embedded vision applications [22]. Therefore, memory usage should be seriously taken into consideration. It takes the advantage of the Xception network architecture to solve this issue by basing its architecture on the same depth-wise separable convolutions. There are multiple versions of MobileNets, out of which MobileNet-V1 and MobileNet-V2 are the most used ones in this field. MobileNet-V1 uses 13 blocks, being composed of depth-wise separable convolution and point-wise convolution. MobileNet-V2, as an improved version of MobileNet-V1, uses 17 bottleneck blocks, each of them composed of a point-wise convolution, depth-wise convolution, and an expansion module. The expansion module would have the responsibility to allow the network to learn a richer function by increasing the size of the representation within the bottleneck block. The point-wise convolution would have the responsibility of down projecting the data to reach its size. MobileNet-V2 also introduced residual connections around bottleneck blocks.

DenseNet-201, first introduced in [23], is also a commonly used convolutional neural network in recent research papers related to skin lesion detection systems. The authors used the concept of densely connected layers, and thus these networks have the advantage of each layer being fed with additional inputs from all the other preceding layers and providing its feature map to all subsequent layers [3]. There are multiple dense network variants, such as DenseNet-121, DenseNet-161, DenseNet-169 and DenseNet-201.

**Figure 4:**
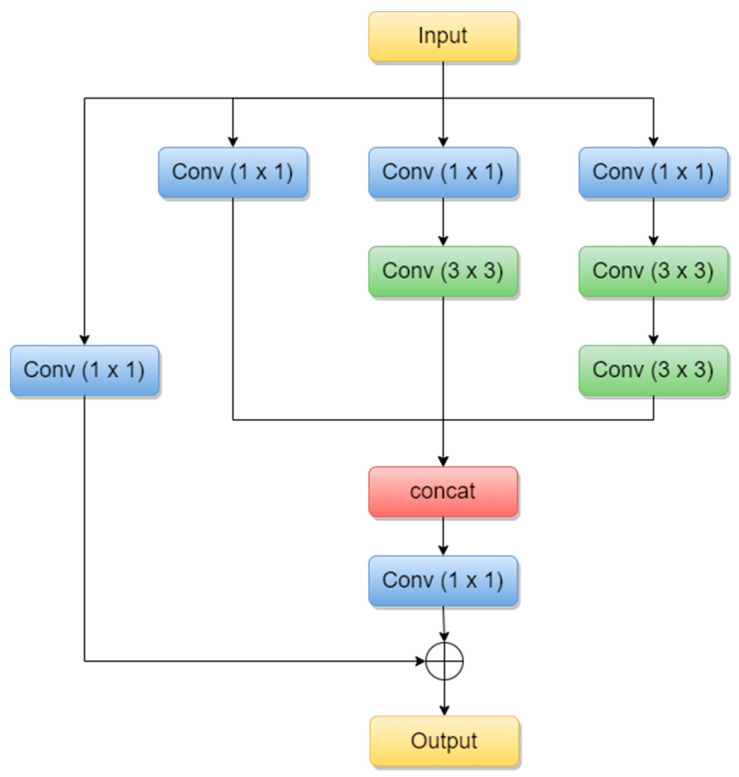
Residual-Inception block (adapted from [25]).

Inception-ResNets are CNNs that combine the Inception architecture with the residual connections [24]. Residual connections were introduced to solve the vanishing gradient problem when it comes to deeper models. The combination of the Inception module with residual connections is called the Residual-Inception block and it can be seen below in Figure 4.

Inception-ResNet-V2 is 164 layers deep and the version we are using in our experiments is pre-trained on the ImageNet database, able to classify images into 1000 object categories such as keyboard, animals, etc.

### 3.3. Proposed Collective Intelligence-Based System

The scope of this paper is to propose a multi-network system (named Collective Intelligence-based System, CIS) based on the decision fusion of each of the involved networks and to obtain better performances in terms of accuracy as compared with the accuracy of each individual network. In the literature, this concept is also called the “ensemble” model, and it was demonstrated that this model in general performs better. Thus, our system involves multiple convolutional neural networks, which were trained and validated on the HAM10000 dataset. The experiments with them in an offline mode identify the performance of each and predicts the final diagnosis based on the decision fusion of the individual models. The functional key of CIS is a Decision Matrix based on adaptive weights. To simplify the writing further, the notations in Table 1 are considered.

The operation of the system involves four phases (learning, validation of individual networks, matrix [W] establishment, performance testing):

1. In the learning phase (3501 images) the configuration (parameters) of each neural network is established and remains fixed.
2. The validation phase for each network uses fewer images (1499) than the learning phase (if we used the same images we would get “better performance”, which would be false). We used this phase to choose the networks (as smart individual classifiers) by considering the good individual performances.
3. In the phase of establishing the weight matrix [W], which is important in the structure of the Collective Intelligence-based System, we used other images (3501 images), different from the two phases. Intuitively, we considered that this number, like the one in the learning phase, establishes the elements in [W] more correctly than if we considered the images from phases 1 and 2.
4. The rest of the images in H10000 (1514 images) will be used as a test or operating images, with all system parameters being set in the previous phases.

In the phase, the weights of each individual CNN are evaluated for each class of the investigated lesions. Therefore, we propose the weights to be calculated as follows:

(1) $$W_{i,j}=\frac{N\_poz_{i,j}}{N\_tot_{i,j}}$$

**Figure 5:**
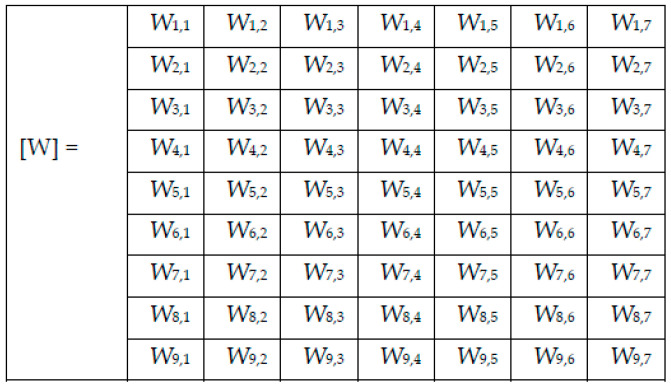
Weight matrix [W].

In (1), $W_{i,j}$ represents the computed weight for the network NN_i_ and the skin lesion of the class *C_j_*, $N\_poz_{i,j}$ represents the number of images correctly classified as skin lesion of type *C_j_* by the network NN_i_, and $N\_tot_{i,j}$ represents the total number of images for skin lesion of type *C_j_* used in testing network NN_i_. After applying (1) on the results found for each confusion matrix presented in the experimental results section above, the weights presented in Figure 5 were computed.

Thus, for a skin lesion image as input, the individual decision block NN_i_ will give a value of the decision *d_i_*_,*j*_ = 1 if the input image belongs to the class *C_j_* and *d_i_*_,*k*_ = 0 in rest. The decision of NN_i_ for the final block CDB (Collective Intelligence Block) will have a confidence (considered as entry in CDB) of $W_{i,j}$.

Once we computed the weights, we calculated each of the network’s weighted decisions as (2):

(2) $$D_{i,j}=W_{i,j}d_{i,j},\;i=1,\;2,\ldots ,\;9;\;\;j=1,\;2,\ldots ,\;7$$

**Figure 6:**
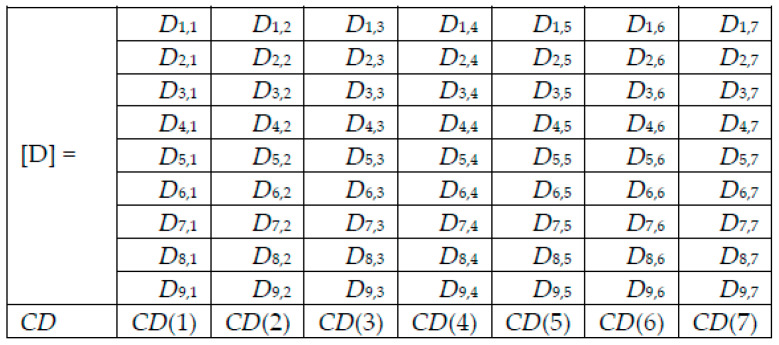
Decision matrix [D] and collective decision (CD).

For each image to be tested/predicted, we obtain a weighted decision as an output of each network, and thus, for each skin lesion image to be classified, we obtained the following array of 9 rows (networks) and 7 columns (possible decisions) (Figure 6). Note that only one element on a row is different to 0.

Adding the elements on column *j* of *D_i,j_* gives the value *CD*(*j*), *j* = 1, 2, …, 7, from the collective decision row.

The decision *D_m_* for an image *I* to be a *C_m_* lesion is made by CDB based on the maximum of (4).

(3) $$CD\left(j\right)=\overset{9}{\underset{i=1}{\sum }}D_{i,j}$$

(4) $$I⋲C_{m}\;\;if\;CD\left(m\right)=\max\left(CD\left(j\right)\right),\;j\in \left\{1,2,3,4,5,6,7\right\}$$

**Remark** **1.** *The weight matrix [W] is established in the special phase 3 and remains constant. Instead, the values of the decision matrix [D] and the collective decision CD are changed for each tested image by selecting from the matrix W the corresponding values, according to the algorithm described (2).*

**Figure 7:**
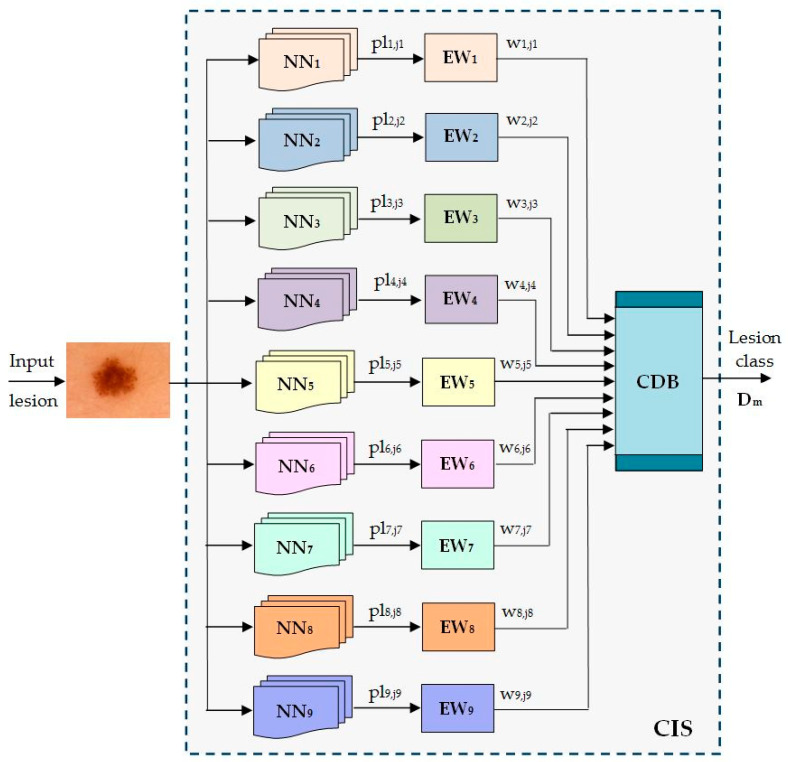
Proposed ensemble model for skin lesion diagnosis.

The scheme of the Collective Intelligence-based System (CIS) is presented in Figure 7. The significance of the notations in the figure is the following:

NN_i_—neural networks implied as individual classifiers’

pl_i,ji_—predicted lesion by the NN_i_ to belong to class *C_j_*;

EW_i_—Establishing Weight module for NN_i_;

*w*_*i*,*ji*_—the weight associated with NNi when a lesion from the class *C_j_* is predicted;

D_m_—the decision that the input lesion belongs to class *C_m_*;

CDB—Collective Decision Block;

CIS—Collective Intelligent System.

The system implementation was done using Matlab and its Deep Learning Toolbox. Each of the convolutional networks was trained using a PC without GPU, and so there was only a single CPU. Of course, training time could have been less when using GPU.

All pre-trained deep learning toolbox models were downloaded from the Matlab “File Exchage” source and were trained with custom configurations and datasets.

## 4. Experimental Results

In this section, we present the performances of each of the individual CNNs involved in the proposed ensemble model. As we mentioned, our purpose was to cover the most important CNN families and to obtain a final decision based on collective intelligence. All these networks have both advantages and disadvantages and of course, there is no ideal system. However, by combining their abilities, we would for sure be able to obtain better performances. It is the same as in the case of a team. One team could have a key player, solving the most complex tasks; however, other team members could also have an important impact on the same team, each of them having different pieces of knowledge which might not be covered by the key player. Therefore, each opinion matters based on its individual performance. For instance, the opinion of the team key player would matter more than the opinion of another team member with less performance. In our case, this is of course covered by the wights computed based on individual performances. As specified in Chapter 3, we chose to involve the following convolutional neural networks: AlexNet, GoogLeNet, GoogLeNet-Places365, ResNet-50, ResNet-101, Xception, MobileNet-V2, DenseNet201, and InceptionResNet-V2.

For the HAM10000 dataset, we had to build a small script that automatically reads the metadata file (containing the name of each image, associated with a dedicated label/diagnosis), and creates the folders with the name equal to the label (if they were not created before), and groups all images in their associated folders. Thus, we ended up obtaining seven folders, each representing a specific label and containing the specific images. The images inside those folders did not have the needed sizes, so they could not be fed directly to the networks. We used an augmented image datastore to resize the images according to the expected sizes by each network before passing them as input.

For the training options configuration, the following parameters were used:

- Mini batch size: 8 or 10 (depending on the network).
- Max epochs: 6 or 10 (depending on the network).
- Validation frequency: 10 or 100 iterations (depending on the network).
- Initial learn rate: 1 × 10^−4^.
- Learn rate drop factor: 0.1 on each 3 epochs (used only for DenseNet201 and Xception).
- Execution environment: parallel (used only for DenseNet201 and Xception) to use multiple workers in parallel to possibly speed up training time.

The images for learning, validation, and testing are taken from the two parts of the H10000 dataset. Because the image numbers per class are imbalanced, the data augmentation for non-nv was used in the training phase (Table 2).

**Figure 8:**
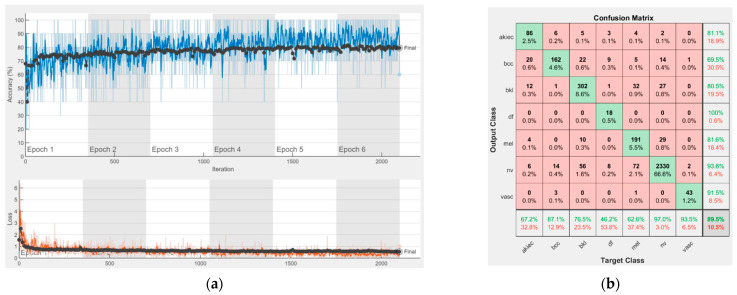
Results using AlexNet. (**a**) Accuracy and loss, (**b**) Confusion matrix.

**Figure 9:**
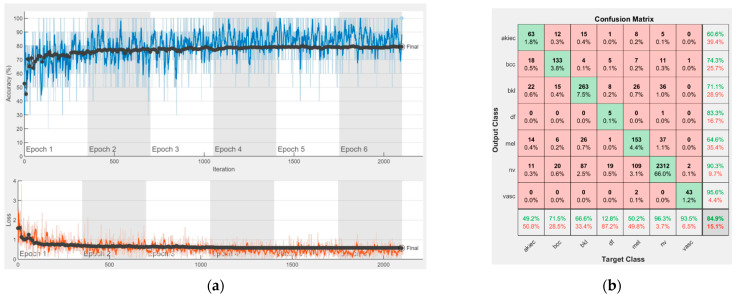
Results using GoogLeNet pre-trained on ImageNet, (**a**) Accuracy and loss, (**b**) Confusion matrix.

**Figure 10:**
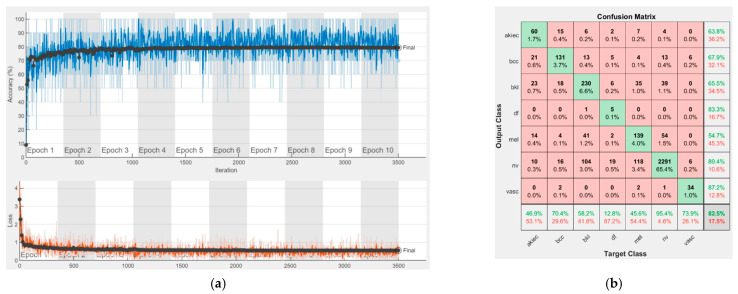
Results using GoogLeNet pre-trained on Places365, (**a**) Accuracy and loss, (**b**) Confusion matrix.

**Figure 11:**
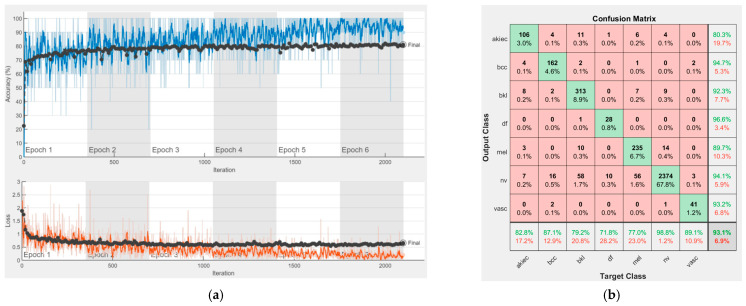
Results using ResNet-50, (**a**) Accuracy and loss, (**b**) Confusion matrix.

**Figure 12:**
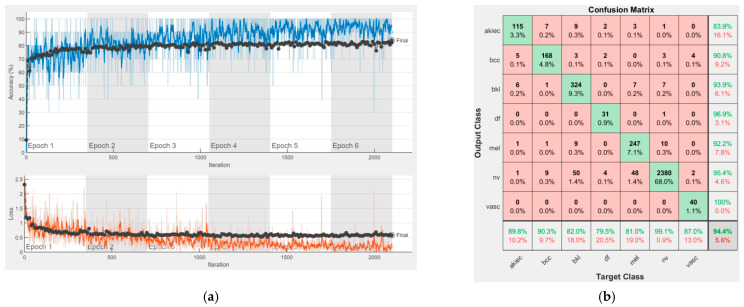
Results using ResNet-101, (**a**) Accuracy and loss, (**b**) Confusion matrix.

**Figure 13:**
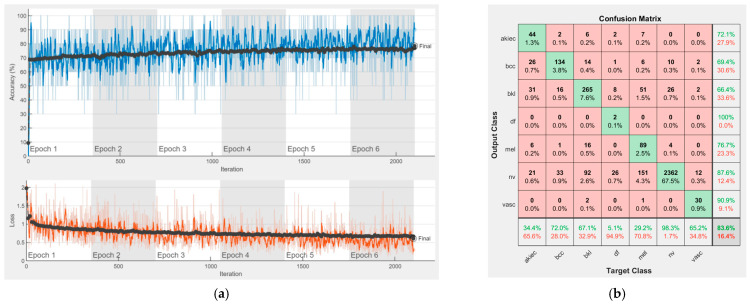
Results using Xception, (**a**) Accuracy and loss, (**b**) Confusion matrix.

**Figure 14:**
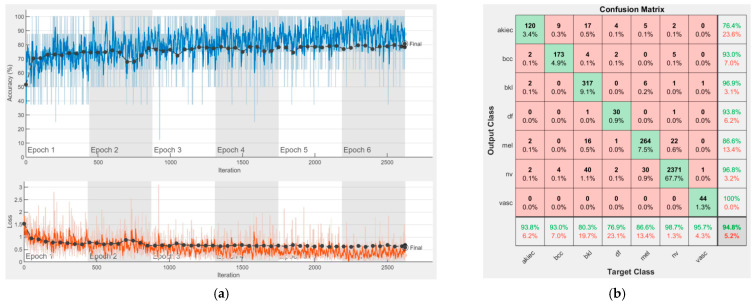
Results using MobileNet-V2, (**a**) Accuracy and loss, (**b**) Confusion matrix.

**Figure 15:**
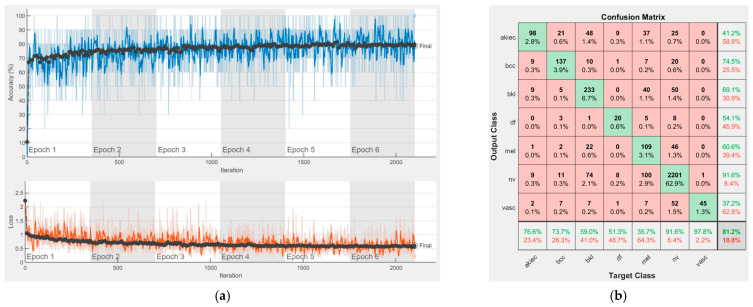
Results using DenseNet-201, (**a**) Accuracy and loss, (**b**) Confusion matrix.

**Figure 16:**
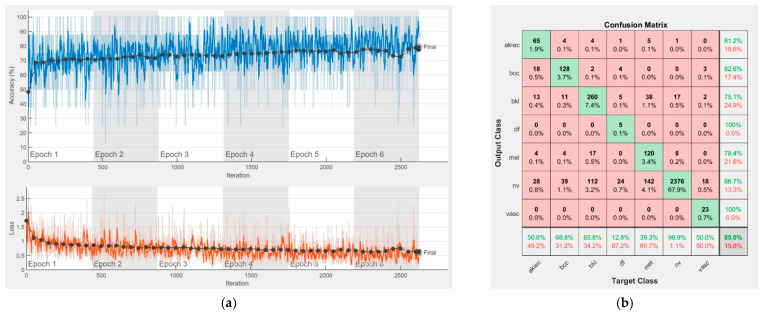
Results using InceptionResNet-V2, (**a**) Accuracy and loss, (**b**) Confusion matrix.

Figure 8, Figure 9, Figure 10, Figure 11, Figure 12, Figure 13, Figure 14, Figure 15 and Figure 16 represent experimental results obtained on the H10000 dataset for each individual CNN involved in the proposed skin lesion classification system. The experiments performed in terms of training are based on 70% of the first part of the HAM10000 dataset. The other 30% of the first part of the dataset was used as an individual validation to choose the neural networks by considering the performance (accuracy). In our case, we considered a threshold of 75% accuracy. About 70% of the second part of the HAM10000 dataset (3501 images) is then used to establish the weight matrix [W].

We used the first part of HAM10000 for singular networks analysis. The reason for choosing 70% of the first part of the HAM10000 dataset for training and the remaining 30% for individual neural network validation is because such a proportion is recommended. We used the second part of HAM10000 for CIS parameter selection and fixation ([W] and [D] matrices) and to experiment with the real operation of the CIS. The reason for choosing 70% of the second part of the HAM10000 dataset was to use a large number of images, other than those in the previous phases, for determining with more precision the elements of the [W] matrix. We kept the remaining 30% of the second part for the operation phase (testing) on new, unused images; the system has, in this case, a fixed structure (parameters).

**Figure 17:**
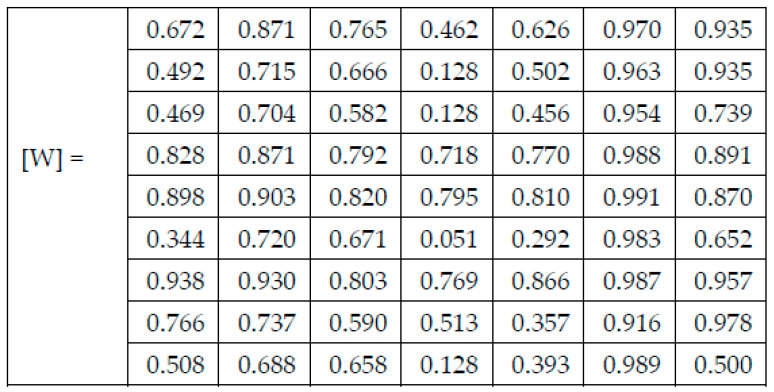
The resulting Weight matrix [W].

Each figure has two sections, (a) and (b). In the sections (a) graphics with training and validation results over time, based on configured epochs/iterations, are presented. On the horizontal axis, the number of epochs and iterations are marked, while on the vertical axis, the accuracy and loss are marked. Blue color and red colors represent the accuracy and loss applied for the training dataset, while black represents the accuracy and loss applied for the validation dataset. Sections (b), for each figure, represent the confusion matrix obtained for individual networks on 70% of randomly selected images from the HAM10000, part 2, dataset. Basically, from the confusion matrix, we can determine the behavior in terms of the final prediction, for each network, and for each class to implement the [W] matrix. We will further use the information from each confusion matrix in our proposed system. After applying (1) to the results obtained for each confusion matrix (based on the testing dataset), we obtained the weight matrix presented in Figure 17. The same information could be found directly in the confusion matrix. Table 3 presents the accuracy obtained during training and validation phases, together with the percentage of the correct classification obtained for each of the classes during the establishing [W] phase.

It can be seen that top-performer networks are based on residual connections. We found that the accuracy grew as we used deeper networks with residual blocks. Therefore, for MobileNet-V2 we obtained a validation accuracy of 80.59%, for ResNet-50 81.12% and for ResNet-101 83.99%.

The elements of [W] are obtained from Table 3, the correct classification section. The other 30% of images from H10000, the second part, are used to test the functionality (Table 4) and performances (Table 5) of CIS. Thus, Table 4 and Table 5 are based on experiments made in phase 4 (test phase) by using images from the test set. As previously mentioned, the elements of the D matrix, which is used only in the test (operation) phase for each input image analyzed, are in fact the elements of the W weight matrix (created in special phase 3) and activated by the results of individual networks in this operation. Thus, a line in D (corresponding to the assigned network) contains 0 if the network does not indicate the class of column and the corresponding element of W if the network indicates the class of that column. D changes with each input image analyzed in the test phase.

**Figure 18:**
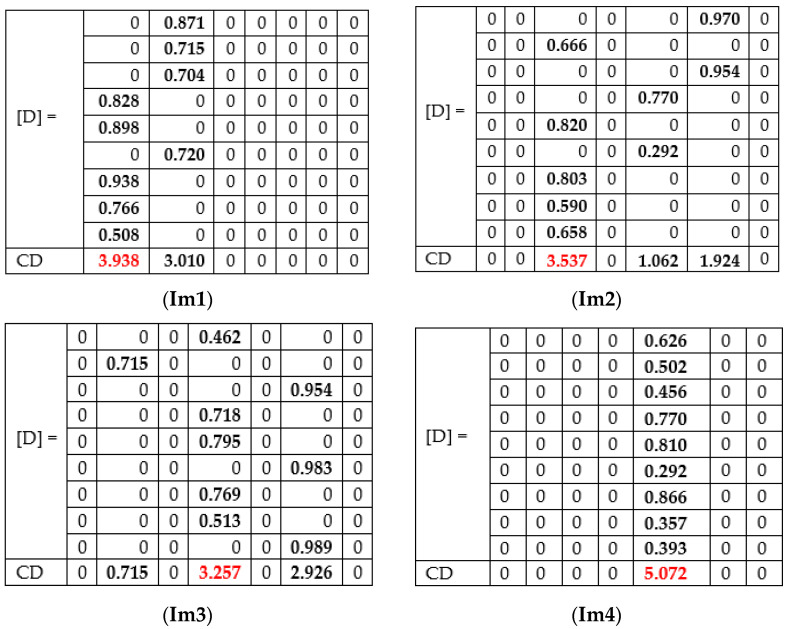
Decision matrices [D] and Collective decision CD for images Im 1, Im 2, Im 3, and Im 4.

Examples of experiments on four randomly selected images (Im 1, Im 2, Im 3, and Im 4) are presented in Table 4. The final decision taken by CDB is based on Decision matrix D, which is calculated for each input image (Figure 18). The decision is taken if CD is at a maximum (red color).

For the first image (Im1), the correct prediction of akiec is obtained, taking into consideration some of the networks (AlexNet, GoogLeNet, GoogleNet-Place365, and Xception) wrongly predicted as bcc. By checking the weighted decision matrix proposed in the Weight matrix, we can see that, even if some of the networks predicted the final decision to be bcc, they are not equally taken into consideration, since other networks with higher computed weights for akiec have a greater priority.

In the case of Im 2, there are three possible outcomes in terms of individual networks (nv, bkl, and mel). In general, all networks perform well in the nv class, considering the high number of images within that class that the individual networks learned. According to the computed weighted matrix W, the minimum weight for nv is 0.916, computed for DenseNet-201, while the maximum weight is 0.991, which is computed for ResNet-101. In the case of the bkl, the rest of the votes were obtained. Thus, it is demonstrated that collective intelligence could bring lots of benefits when it comes to the final prediction, even if some of the networks predict a class with the highest weights. An example of a final prediction by correction was bkl, which was given by our model.

In terms of the third image, our model correctly predicted df, while GoogLeNet predicted bcc, GoogLeNet-Places365, Xception, InceptionResNet-V2 predicted nv and the others predicted df. Even in this case, where three networks predicted nv with considerable weights, the winner was df, because of the collective approach.

Of course, there are cases in which all networks provide the same prediction. In the case of the Im 4, the prediction was Mel.

Compared to the classical voting system, in which the result is the class voted by the majority, taking Im1 as an example and assuming NN9 would have predicted bkl, then bcc and akiec would have had the same number of votes (4). akiec would no longer have voted for the majority. However, the impact of the four networks for the akiec class is higher (due to the proposed weights) and the final result would have a total score of 3.43 compared to 3.01, the final score for bcc. The decision of the CIS will also be akiec.

## 5. Discussion

To implement the Collective Intelligence-based System, we analyzed the performances of the proposed neural networks as individual intelligence-based classifiers for skin lesion diagnosis. The performances were translated into trusts given to the respective neural networks in the form of weights. Given that the networks have an outstanding performance for various classes of lesions, the weights have been adapted to the classes predicted in the testing phase. Thus, the proposed system is based on a matrix of decisions in which all these particularities are introduced, the final decision being taken on an optimal criterion (maximum value of the sum of weighted decisions). Calculating the average accuracy as a statistical indicator in the operational phase (testing), the comparative table was obtained, Table 5. It is observed that the average accuracy of the proposed system (for all 7 classes) is better by 2.72% to 4.26% than the average accuracy of the individual networks. As we can see in Table 5, we obtained an overall accuracy of 86.71% by using the collective intelligence-based model. The different values of the performances (weights) can be attributed both to the characteristics of the classes and to the imbalance of the number of representatives of the classes.

A new network/classifier can be added to the proposed system, providing that the new obtained system has a better validation performance than the old one. If this does not happen, the network is not considered eligible and cannot be added. However, before adding another individual classifier, it must be individually trained on the same dataset and the individual performances must be analyzed. Once we have its performance, we need to compare it with other individual CNNs involved in the same system and we may choose to replace them with a new one, in case the new one performs better than the old one. The main idea of this paper is to illustrate that the means of combining individual classifiers is important for obtaining better results and not the number of involved networks/classifiers in the system. Of course, once another individual classifier is added and the other is removed, we need to recalculate the weight matrix and take the newly added/removed weights into consideration as well, before classifying new images. It should be noted that the number of neural networks was chosen experimentally by considering similar performances, and that there remains the belief that a larger number of subjective classifiers, as these networks can be considered, can provide a more objective classification overall. However, the number cannot be overstated, as it could unduly increase the complexity of the global system without a remarkable increase in performance. For this reason, in the future we propose an iterative method of choosing neural networks for the composition of the CIS system, analyzing the evolution of performance with each step, which will stop when saturation is reached.

In terms of time performance, the results are given in Table 6. It can be observed that the operating time of the CIS system is dictated by the neural network that has the longest time, respectively IncepetionResNet-V2.

There is no risk in overfitting, since the multi-network system was implemented considering the decision output of each convolutional neural network and by obtaining good results in terms of training experimental results (as can be seen in Figure 8, Figure 9, Figure 10, Figure 11, Figure 12, Figure 13, Figure 14, Figure 15 and Figure 16). Since the overfitting in terms of individual networks was avoided and we combined the decisions of each network by using the proposed weighted decision method to obtain better results, there should be no risk of overfitting.

On the other hand, the new method of deep transfer learning [26] can improve the detection of skin lesions in the case of a lower number of images in the database.

A comparison with similar papers, with systems based on an ensemble of neural networks, is given in Table 7.

The paper illustrates that the combination of multiple convolutional neural networks based on the proposed method represents a means of obtaining better results as compared with the results of each individual network. We would like to point out that a large number of the involved networks in the ensemble do not matter, but rather the means of combining them to obtain better results do. This solution was inspired by real-life scenarios, where, for instance, a second or even a third physician’s opinion matters in terms of a more accurate decision related to a particular disease. In our case, each convolutional neural network could be trained separately in an offline mode and some of them might perform better in the case of disease and some of them might perform better in case of other diseases. Based on individual performances, we can decide on the impact of each for each disease to obtain a more accurate decision. The main reason we used more convolutional neural networks was to experiment with the most used networks in this field for the HAM10000 dataset. Of course, within the experiments, we noticed that there are indeed a small number of networks that do not perform well for some classes; however, according to the proposed method of combining them, they would have less of an impact on the classification for the diseases for which they do not perform well. On the other hand, even networks with less accuracy for some of the classes might have a positive impact when it comes to other classes.

## 6. Conclusions

The paper proposed a system based on the collective intelligence of nine neural networks for the detection and classification of skin lesions into seven classes defined in the H 10,000 database with better statistical performance than each network. As a novelty, flexible weights attached to the networks were used, depending on the class provided. We chose to propose an ensemble model, since we demonstrated that it could achieve better performance than each of the individual networks. We propose having a more adaptive fusion of individual classification decisions rather than a simple voting scheme. Thus, the variable weights will not contribute equally to the final decision but according to the performance of the individual networks to the detection of a certain class. In other words, we propose to associate a specific weight *W*_*i*_,_*j*_, not only with the network NNi, but also with its individual predicted skin lesion class *C_j_*. The decision fusion module takes into consideration all weights and makes the final decision. This model could be easily modified with more networks or other classifiers. However, before doing this, our recommendation would be not only to add new classifiers and compute the new weight matrix, but also to perform an in-depth analysis of both the already involved classifiers and the new classifier(s) and, at some point, to decide to potentially replace the ones performing less with the new one(s). We would like to note that a large number of involved classifiers do not matter, but their individual performances and the customized means of combining them to obtain better results do. Researchers are also encouraged to reuse existing networks, or maybe decommission some and develop their own customized means to combine them for obtaining better results. In terms of used datasets, we chose HAM10000 because of the detailed samples for all seven classes. A disadvantage of the database used is the unbalanced structure of the classes involved. The users could train their own individual classifier(s) using the same or even a smaller number of samples, but with balanced classes, and adapt the proposed system in such a way as to obtain better results.

The number of nine networks was chosen experimentally based on an intuitive criterion that more subjects can make a better collective decision. A performance threshold of 75% has been introduced for each network. As a future direction, we want to introduce a criterion based on the iterative construction of the system, introducing/eliminating networks until saturation is reached (an insignificant increase in performance). Other single intelligent classifiers may also be considered.

## Figures and Tables

**Table 1:** Notations for the neural networks and lesion classes used.

CNN	Notation	Lesion	Classes
AlexNet	NN_1_	akiec	C_1_
GoogLeNet	NN_2_	bcc	C_2_
GoogLeNet-Places 365	NN_3_	bkl	C_3_
ResNet-50	NN_4_	df	C_4_
ResNet-101	NN_5_	mel	C_5_
Xception	NN_6_	nv	C_6_
MobileNet-V2	NN_7_	vasc	C_7_
DenseNet-201	NN_8_		
InceptionResNet-V2	NN_9_		
Collective Intelligence Block	CDB		

**Table 2:** The images used per class with augmentation (only training phase) for the non-nv.

Class	Training Each NN (3501 Images from the First Part)	Validation for Selection of Each NN (1514 Images from the First Part)	Establishing the Weight Matrix (3501 Images from the Second Part)
akiec	268	114	128
bcc	326	139	186
bkl	535	229	395
df	179	79	39
mel	445	190	305
nv	1562	669	2402
vasc	186	79	46
TOTAL	3501	1499	3501

**Table 3:** The performances obtained for the singular networks.

Network	Accuracy (%)		Correct Classification (Phase 3) (%)							
	Training (Phase1)	Validation (Phase 2)	akiec	bcc	bkl	df	mel	nv	vasc	Total
NN_1_	93.89	79.65	67.2	87.1	76.5	46.2	62.6	97.0	93.5	89.5
NN_2_	87.55	79.45	49.2	71.5	66.6	12.8	50.2	96.3	93.5	84.9
NN_3_	84.15	79.45	46.9	70.4	58.2	12.8	45.6	95.4	73.9	82.5
NN_4_	98.46	81.12	82.8	87.1	79.2	71.8	77.0	98.8	89.1	93.1
NN_5_	98.91	83.99	89.8	90.3	82.0	79.5	81.0	99.1	87.0	94.4
NN_6_	83.58	78.45	34.4	72.0	67.1	5.1	29.2	98.3	65.2	83.6
NN_7_	94.80	80.59	93.8	93.0	80.3	76.9	86.6	98.7	95.7	94.8
NN_8_	83.06	78.59	76.6	73.7	59.0	51.3	35.7	91.6	97.8	81.2
NN_9_	85.03	79.39	50.8	68.8	65.8	12.8	39.3	98.9	50.0	85.0

**Table 4:** Experimental results in the case of four randomly selected images.

Selected Images Networks	Im1 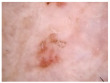	Im2 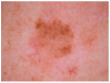	Im3 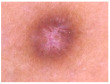	Im4 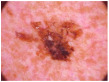
NN_1_	bcc	nv	df	mel
NN_2_	bcc	bkl	bcc	mel
NN_3_	bcc	nv	nv	mel
NN_4_	akiec	mel	df	mel
NN_5_	akiec	bkl	df	mel
NN_6_	bcc	mel	nv	mel
NN_7_	akiec	bkl	df	mel
NN_8_	akiec	bkl	df	mel
NN_9_	akiec	bkl	nv	mel
**CDB**	**akiec**	**bkl**	**df**	**mel**

**Table 5:** The proposed system with the collective decision and individual network performances in order of increasing performance—test phase.

Network	Accuracy
Xception	78.45%
DenseNet-201	78.59%
IncpetionResNet-V2	79.39%
GoogLeNet-Places365	79.45%
GoogLeNet	79.45%
AlexNet	79.65%
MobileNet-V2	80.59%
ResNet-50	81.12%
ResNet-101	83.99%
**Proposed CIS**	**86.71%**

**Table 6:** The proposed system with collective decision execution times for 30 randomly selected images in order of the increasing time.

Network	Execution Time (Seconds)
AlexNet	0.3703
MobileNet-V2	0.5800
GoogLeNet	0.5998
GoogLeNet-Places365	0.6240
ResNet-50	1.0424
DenseNet-201	1.5299
ResNet-101	1.6435
Xception	2.7166
IncepetionResNet-V2	3.2090
**Proposed CIS**	**3.2950**

**Table 7:** A synthetic comparison with the results in similar papers.

Paper	Description	Our Differences
[27]	▪ 4 CNN fused decisions ▪ Classification for 3 classes (nevus, melanoma, and seborrheic keratosis) ▪ Simple majority voting model (accuracy of 86.6%) ▪ Sum of the maximal probabilities model (accuracy 86.9%)	▪ 9 CNN fused decisions based on adaptive weights ▪ Classification for 7 classes including df with poor performance) ▪ Our model with 9 networks has accuracy of 86.71% for 7 classes
[15]	▪ Classification includes melanoma ▪ 3 CNNs used for feature extraction ▪ Datasets (PH2-200 + ISIC-MSK-225 + ISIC-UDA-557 + ISBI-2017-2750) ▪ less than 5000 images	▪ Classification includes 7 classes (all HAM 10,000 classes) ▪ 9 CNNs used ▪ 10015 images
[16]	Voting system	Combined output/intelligence by weighted decisions per each class
[10]	▪ Combined decisions based on individual network/classifier overall accuracy ▪ 1 custom CNN + 3 CNNs + 1 feature-based classifier ▪ Total number of 300 images (100 from PH2 and 200 from ISIC 2019) ▪ 2 Classes (melanoma and nevus) ▪ Training accuracy for ResNet-101 was 90% and validation accuracy was about 70%	▪ Combined decisions based on individual network performance for individual disease ▪ 9 CNNs ▪ 10015 images ▪ 7 classes classification ▪ Training accuracy for ResNet-101 was 98.91% and validation accuracy was 83.99%
